# Nuclear actin switch of the INO80 remodeler

**DOI:** 10.1093/jmcb/mjy083

**Published:** 2018-12-18

**Authors:** Jun Wu, Yimin Lao, Bing Li

**Affiliations:** 1 Department of Biochemistry and Molecular Cell Biology, Shanghai Jiao Tong University School of Medicine, Shanghai, China; 2 Department of Laboratory Medicine, Shanghai General Hospital, Shanghai Jiao Tong University, Shanghai, China

Eukaryotic DNA is wrapped in nucleosomes, which impede the access of transcription factors and regulatory proteins to template DNA. Chromatin remodelers utilize the energy from ATP hydrolysis to drive histone movement relative to nucleosomal DNA and nucleosome editing. Thus, they play critical roles in transcription, DNA replication, and damage repair, and their dysfunctions are often associated with diseases including cancers (Klages-Mundt et al., 2018). Chromatin remodelers can be generally categorized into INO80, SWI/SNF, CHD, and ISWI subfamilies, which share conserved catalytic ATPase-translocase motors. However, how other auxiliary components of these multi-subunit machinery control their genomic recruitment and actions of DNA translocation remains as a major challenge of the field.

Recent structural and biochemical studies provide ground-breaking insights on how multi-subunit chromatin remodelers engage with nucleosomes and their acting mechanisms (Aramayo et al., 2018; Ayala et al., 2018; Knoll et al., 2018; Willhoft et al., 2018). A common theme emerged from these studies is that a remodeler complex tends to make multiple contacts with nucleosomes in order to properly couple its ATPase activity with nucleosome mobilization activities: (i) the motor domains of many remodelers share a common nucleosomal binding site at the superhelical location +2 (SHL+2), which locates two helical turns away from the nucleosome dyad axis (Aramayo et al., 2018; Ayala et al., 2018; Knoll et al., 2018; Willhoft et al., 2018); (ii) closely related INO80 and SWR1 both use an Arp module (Arp5/Ies2 in INO80; Arp6/Swc6 in SWR1) to grab DNA at the opposite sites of nucleosome comparing to the motor domain and also bind to the acidic patch of the histone globular domain (Ayala et al., 2018; Eustermann et al., 2018; Willhoft et al., 2018). These contacts provide an anchor point for remodelers to harness torsional tension generated through DNA translocation to disrupt histone–DNA interactions (Clapier et al., 2017), which in turn trigger subsequent nucleosome mobilization and/or potential histone editing (Willhoft et al., 2018).

**Figure 1:**
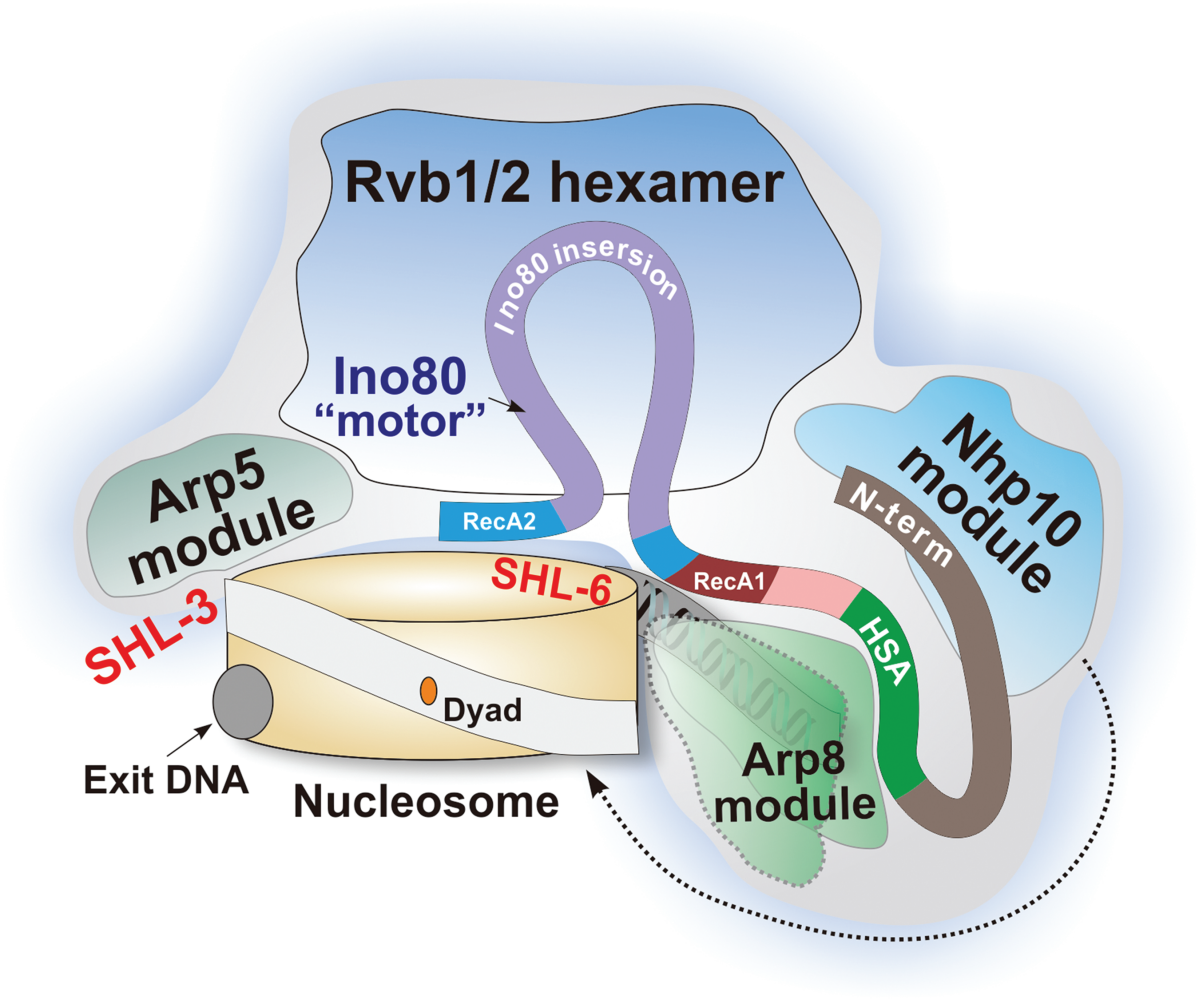
A working model of the INO80 complex interacting with a mono-nucleosome. The green shape circled with a dash line represents the second binding conformation of Actin/Arp8 module.

Using Cryo-EM and subunit deletion analysis of the native yeast INO80 complex, the current structural study by Zhang et al. (2018) revealed exciting new insights on INO80 submodule assembly and a key functional switch that coordinates its remodeling activity. A working model of how the INO80 complex interacts with nucleosomes emerged from these studies is summarized in Figure 1. The catalytic subunit Ino80 serves as a center scaffold to nucleate complex assembly: its insertion motif binds to Rvb1/2 hexamer module, which links the Arp5 module that contacts nucleosomes at the SHL−3 position; unlike SWR1 and other Snf2-like translocase domains, the motor domain of Ino80 targets to SHL−6; the helicase-SANT associated domain (HSA) of Ino80 forms a stable submodule with Arp8/actin/Apr4, which binds to the linker DNA. Unlike studies based on the INO80 core complex, the intact native INO80 complex used by Zhang et al. (2018) allowed the first visualization of the Nhp10 module, which is responsible for high affinity nucleosome interaction but not ATPase activity. This was achieved through comparing density differences between various subunit deletion mutant complexes and cross-referencing subunit interaction information from cross-linking mass spectrometry (CX-MS) analysis.

Most intriguingly, Zhang et al. (2018) reported for the first time two conformational states of the actin/Arp8 submodule upon its contacting nucleosomes, which indicates that nuclear actin (N-actin) can act as a ‘switch’ to regulate remodeler activities. Actin is one of the most conserved and abundant proteins in eukaryote cells. However, functions of N-actin have been controversial for decades since its first discovery in the 1960s. N-actin and actin-related proteins (Arps) have been identified as stoichiometric components of many chromatin remodelers such as HDAC, SWI/SNF, and INO80 (Klages-Mundt et al., 2018). However, unlike its cytosolic polymerized counterpart, the N-actin of INO80 exists as a monomer (Klages-Mundt et al., 2018), implying its distinguished functions from traditional roles. Arp8 is the key organizer for this actin/Arp module. Consistent with previous structural and biochemical results (Brahma et al., 2018; Knoll et al., 2018), Zhang et al. (2018) also found that actin/Arp8 module interacts with extra-nucleosomal DNA. Importantly, 3D reconstruction of this submodule and mono-nucleosomes bearing a 30-bp DNA linker at both ends showed that INO80 binds to nucleosomes at 1:1 stoichiometry in an ATPase activity-independent manner. The actin/Arp8 module displays two distinct conformational states: in the state I, linker DNA only contacts the Arp8 peripheral region; in the more intimate binding state II, Arp8 undergoes extensive conformational changes, which allow it to hug exposed histone surface while drawing Arp4/actin module toward the vicinity of nucleosome to make additional contacts (Figure 1). These two states may reflect the ability of the actin/Arp8 module to stabilize momentary unwrapped histone–DNA contacts during DNA translocation, thereby promoting the wave-ratchet mode of remodeling (Clapier et al., 2017) and/or its ability to sense the linker DNA length.

Since the actin/Arp8 module in INO80 is evolutional conserved, exploring the structural and biochemical properties of this module will advance our understanding of mechanisms by which nuclear actins in other chromatin remodelers facilitate their functions. Indeed, by studying the cryo-EM of chromatin remodeler SWI/SNF, the same group found that the Arp module (Arp7, Arp9, and Snf2^HSA^) of SWI/SNF can also contact linker DNA, which in turn brings the nucleosome substrate to the vicinity of catalytic Snf2 ATPase domain. This important work also raised several interesting questions for future studies. How do different actin/Arp modules dictate the recruitment of various remodelers to chromatin? The binding of the Arp5 module to acidic patch of H2A/H2B relies on the ability of Arp8 module binding to linker DNA (Brahma et al., 2018). How do these modules coordinate with each other within INO80? Future structural studies of intact complex interacting with nucleosomes and biochemical/biophysical dissection of dynamic actions between native complex and cognate substrates will be crucial to answer these questions and advance our understanding of the roles of these remodelers in physiological and pathological conditions. *[This work was supported by the National Key R&D Program of China (2018YFC1004500) and the National Natural Science Foundation of China (31872817 to B.L.).]*
